# *Streptococcus suis* meningitis in an elderly man: a case report

**DOI:** 10.3389/fmed.2026.1735413

**Published:** 2026-02-23

**Authors:** Fei Zhou, Yi Zhang, Yong Liu, Yingdong Mou, Jingxia Chen

**Affiliations:** 1 Department of Emergency, Affiliated Hospital of Shandong Second Medical University, Weifang, China; 2 Department of Neurology, Affiliated Hospital of Shandong Second Medical University, Weifang, China

**Keywords:** intrathecal injection, meningitis, MetaCAP, *Streptococcus suis*, vancomycin

## Abstract

**Background:**

*Streptococcus suis* is a zoonotic pathogen that resides in pigs. It can be transmitted to humans through several routes, including contact with sick or carrier pigs via broken skin or mucous membranes and consumption of undercooked pork products. *Streptococcus suis* often causes severe clinical symptoms such as meningitis, sepsis, and shock.

**Case presentation:**

A 66-years-old male butcher was admitted to the hospital with a sudden high fever and disturbance of consciousness, and he remained in a state of persistent restlessness. The neurological examination findings were as follows: he was poorly cooperative with the examinations of higher cortical functions and cranial nerves, uncooperative with the examination of limb muscle strength, and unable to cooperate with the examinations of sensation and ataxia. He presented with nuchal rigidity, with a distance of four finger breadths between the chin and chest, and Kernig’s sign was positive. The patient was diagnosed with *Streptococcus suis* meningitis based on the results of Metagenomic Capture sequencing, cerebrospinal fluid culture, and blood culture. Considering the patient’s critical condition, he had received empirical treatment with cephalosporin in the previous hospital, but the therapeutic effect was not satisfactory. Moreover, in this region, there is a phenomenon of decreased sensitivity in *Streptococcus pneumoniae* to penicillin and third-generation cephalosporins. Therefore, the patient received antibiotic treatment with vancomycin (1 g) intravenously every 12 h. Concurrently, he was administered mannitol to reduce intracranial pressure and ulinastatin for anti-inflammatory effects and immune enhancement. Subsequently, vancomycin 20 mg was administered by intrathecal injection. The patient’s condition improved, and he was discharged from the hospital. There was no special discomfort during follow-up.

**Conclusion:**

This case report describes the diagnosis and treatment process of *Streptococcus suis* meningitis. It proposes an antibiotic treatment plan centered on vancomycin. Intrathecal injection of antibiotics may provide an effective treatment option for severe patients and offer a treatment choice for drug-resistant bacterial infections in the central nervous system. It was also pointed out that Metagenomic Capture sequencing can reduce host gene interference and increase the detection rate of pathogens. This case aims to enhance clinicians’ understanding of the disease and provide a reference for early identification and standardized treatment.

## 1 Introduction

*Streptococcus suis* is a significant zoonotic pathogen worldwide. The main transmission routes for human infection are occupational exposure, mostly through contact with the blood, urine, feces, saliva, and other body fluids of infected pigs with broken skin or mucous membranes, or by handling contaminated pork products (1). A small number of patients develop infections by consuming pork or pork products from infected pigs that were not thoroughly cooked (2, 3). *Streptococcus suis* infection can cause sepsis, meningitis and toxic shock syndrome, etc., (4, 5). *Streptococcus suis* meningitis is a severe infectious disease of the central nervous system that is associated with high fatality and disability. It presents with acute onset and rapid progression, often resulting in severe sequelae, which presents considerable challenges for early clinical diagnosis and treatment. *Streptococcus suis* meningitis initially presents with typical symptoms, such as high fever, severe headache, projectile vomiting, and stiff neck (3). As the disease progresses, it is highly likely to develop complications such as permanent hearing damage, visual impairment, and epileptic seizures (6). Early diagnosis of this disease not only relies on typical clinical symptoms and epidemiological history, but also on cerebrospinal fluid (CSF) bacterial culture, which is the gold standard for detecting *Streptococcus suis* (7). However, false negatives may occur if the patient has already received antibiotic treatment in the early stage. And this test requires fresh samples, strict aseptic operations, and professional laboratory conditions, making it difficult to popularize in some developing countries. In addition, with the extensive use of antibiotic, the problem of *Streptococcus suis* resistance has become increasingly prominent. Relevant research reports suggest that the sensitivity of some strains to penicillin and cephalosporin has declined, and multidrug-resistant strains have emerged, which limits the efficacy of traditional treatment plans (8, 9). Vancomycin, a glycopeptide antibacterial drug, exerts its effects by inhibiting the synthesis of bacterial cell walls (10). Its mechanism of action has no cross-resistance with β-lactam drugs and can still maintain strong antibacterial activity against drug-resistant *Streptococcus suis*.

This article reports a typical case of *Streptococcus suis* meningitis, detailing its clinical features, diagnostic process, treatment plan and prognosis. This case was recorded and strictly carried out in accordance with the internationally recognized CARE checklist (Supplementary material).

## 2 Case presentation

A 66-years-old male patient was admitted to the emergency department at 23:35 due to a 2-days history of fever and an 8-h history of irritability. Two days before admission, the patient experienced an unexplained fever. The highest body temperature reached 39.9°C. He also experienced limb tremors, chest tightness, shortness of breath, and occasional headaches. Eight hours before admission, the patient experienced a recurrence of fever, accompanied by disturbance of consciousness, irritability, and uncooperative verbal communication. The patient received treatment at another hospital, which included an intravenous administration of parecoxib sodium, an intravenous infusion of compound mannitol, and antibiotic treatment with cephalosporin (specific type unknown). Although there was a slight reduction in the body temperature, the patient’s restlessness persisted. Consequently, the emergency department admitted the patient to the Emergency Intensive Care Unit with a preliminary diagnosis of “possible intracranial infection.” The patient had a prior history of good health with no systemic diseases. There was no family history of similar diseases or genetic disorders.

Physical examination at admission revealed the following: body temperature, 36.0 °C; pulse rate, 99 beats per minute; respiratory rate, 26 breaths per minute; and blood pressure, 138/74 mmHg. The patient weighed 85 kg. An elderly male in an agitated state with poor mental status, showed poor cooperation during physical examination. He had a Glasgow Coma Scale score of 12 upon admission (E4V3M5). No obvious abnormalities were observed in the head, face, eyes, ears, nose, or throat. No obvious abnormalities were found on physical examination of the heart, lungs, or abdomen. On neurological examination, the examination of higher cortical functions and cranial nerves showed poor cooperation. The assessment of limb muscle strength was uncooperative, while muscle tone was normal and tendon reflexes were present. Bilateral pathological reflexes were not observed. The examinations of sensation and ataxia could not be completed because of poor cooperation. Nuchal rigidity was present, with a chin-to-chest distance of four fingerbreadths, and Kernig’s sign was positive.

The patient showed marked restlessness after admission. Intramuscular injection of a high dose of diazepam had a poor effect. The patient also had excessive sputum production and low blood oxygen saturation. Therefore, endotracheal intubation and ventilator-assisted ventilation were performed. In terms of treatment, the patient was administered acyclovir for antiviral therapy, meropenem 2 g intravenously every 8 h for antibiotic treatment, compound mannitol for intracranial pressure reduction, and ulinastatin for anti-inflammatory treatment and immune enhancement. The results of the first blood test (1 h after admission) were as follows (Table 1): Blood routine examination: Leukocyte count (WBC) count 21.73 × 10^9^/L, Neutrophil granulocyte (NEUT%) 92.0%. Procalcitonin (PCT): 11.900 ng/ml. C-reactive protein (CRP): 131.55 mg/L. Interleukin-6 (IL-6): 356.00 pg/ml. No significant abnormalities were observed in biochemical test results. Simultaneously, lumbar puncture was performed. Milky rice water-like CSF was drained, and the intracranial pressure (ICP) of the CSF was measured to be >350 mmH2O (1 mmH2O = 0.0098 kPa). A 30 ml sample of CSF was collected and sent to the hospital laboratory for routine tests, biochemical tests, quantitative detection of *Mycobacterium tuberculosis* DNA, CSF bacterial culture, and drug sensitivity tests. As a standard and essential part of the diagnostic workup for suspected bacterial meningitis, blood cultures were obtained concurrently to identify possible bloodstream invasions by pathogenic bacteria. Given that bacterial culture results would take a long time to be available and might show false negatives due to prior antibiotic treatment, we communicated with the patient’s family and suggested sending the specimen for external testing. The family agreed to participate and actively cooperated. Meanwhile, 4 mL of CSF and 4 mL of blood were sent to the Jinan Jinyu Diagnostics Center for Metagenomic Capture (MetaCAP) High-Throughput Pathogen Nucleic Acid Sequencing to identify the type of pathogenic microorganism. The results were as follows: CSF Analysis: Lactate Dehydrogenase (LDH) 307.0 U/L, Glucose (Glu) 0.28 mmol/L, Chloride 116.8 mmol/L, Adenosine Deaminase (ADA) 3.9 U/L, Protein (Pro) 3.590 g/L, Color: Colorless, Turbidity: Turbid, Total cell count 2,630 × 10^6^/L, Mononuclear cells 20%, Polymorphonuclear cells 80%. Immunoglobulins: CSF Immunoglobulin G 917.0 mg/L, CSF Immunoglobulin A 107.0 mg/L, CSF Immunoglobulin M 38.5 mg/L. Detection of *Mycobacterium tuberculosis*: quantitative determination of CSF *Mycobacterium tuberculosis* DNA: Negative. On the 3rd day after admission, blood MetaCAP High-Throughput Pathogen Nucleic Acid Sequencing revealed *Streptococcus suis*, with 243 detected sequences and a relative abundance of 17.21%. Notably, this MetaCAP assay was only used for pathogen identification and did not detect or report mutations in antibiotic resistance gene. On the day the CSF was collected and sent to the hospital laboratory, the Gram stain results were promptly reported: Gram-positive cocci arranged in chains were observed. On the 5th day after admission, the results of CSF culture and drug sensitivity tests were as follows: *Streptococcus suis* was isolated. The bacterium was resistant to ceftriaxone and cefotaxime, but sensitive to vancomycin, azithromycin, erythromycin, cefepime, clindamycin and levofloxacin.

**TABLE 1:** Laboratory test results.

Parameters		Patient values						Reference range
		First blood draw result	Day 3	Day 5	Day 7	Day 10	Day 13	
Blood routine examination	WBC	21.73 × 10^9/L	15.53 × 10^9/L	9.11 × 10^9/L	13.73 × 10^9/L	14.12 × 10^9/L	9.09 × 10^9/L	3.5–9.5 × 10^9/L
	NEUT%	92%	81.70%	60.70%	55.30%	69.70%	50.40%	40%–75%
IL-6		356 pg/ml	104 pg/ml		13.1 pg/ml		8.04 pg/ml	0–7 pg/ml
CRP		131.55 mg/l	207 mg/l	68.34 mg/l	15.8 mg/l			<6 mg/l
ICP		>350 mmHg		320 mmHg	140 mmHg	150 mmHg		80–180 mmHg
Cerebrospinal fluid analysis	LDH	307 U/L		280 U/L	130 U/L	60 U/L		10–25 U/L
	Glu	0.28 mmol/L		4.05 mmol/L	3.51 mmol/L	3.26 mmol/L		2.50–4.45 mmol/L
	Cl	116.8 mmol/L		124.8 mmol/L	129.4 mmol/L	130.4 mmol/L		120–130 mmol/L
	Pro	3.59 g/L		0.96 g/L	1.55 g/L	0.85 g/L		0–0.4 g/L
	Turb	Turbid		Transparent	Transparent	Transparent		
	Cell count	2,630 × 10^6/L		240 × 10^6/L	120 × 10^6/L	8 × 10^6/L		
Blood culture				*Streptococcus suis*		Negative	Negative	
Cerebrospinal fluid culture				*Streptococcus suis*		Negative	Negative	
MetaCAP			*Streptococcus suis*					

WBC, Leukocyte count; NEUT%, Neutrophil granulocyte; IL-6, Interleukin-6; CRP, C-reactive protein; ICP, intracranial pressure; Glu, Glucose; Pro, Protein; Turb, Turbidity.

Upon further inquiry about the patient’s medical history, it was noted that he worked as a butcher. About 5 days before admission, he consumed unclean pork. The routine biochemical tests of the patient’s CSF were consistent with those for bacterial meningitis. Based on the patient’s clinical manifestations, physical examination, CSF culture, and blood MetaCAP results, the patient was diagnosed with *Streptococcus suis* meningitis. Given the critical condition of the patient, previous treatment in the external hospital involved empirical treatment of cephalosporin drugs, but with poor efficacy. Moreover, there is a prevalence of cephalosporin-resistant *Streptococcus pneumoniae* in this region. Therefore, vancomycin (1 g intravenously every 12 h) was added for antibiotic treatment and dexamethasone for anti-inflammatory treatment. The ventilator was intermittently removed to perform respiratory function exercises. During the hospitalization, the patient experienced recurrent fever with significant fluctuations in body temperature and occasional irritability. Considering the poor control of the infection, vancomycin 20 mg was administered by intrathecal injection on the 6th day after admission. The next day, a lumbar puncture was performed again, and clear and transparent CSF was drained. The patient’s ICP decreased significantly (Table 1) and his consciousness gradually became clear. To assess the control of intracranial infection, we repeatedly rechecked the relevant inflammatory indicators; inflammatory markers such as blood routine examination indicators, IL-6, and CRP showed an overall downward trend (Table 1). In addition, we conducted a lumbar puncture test on the 10th and 13th days to measure the intracranial pressure, which gradually decreased to the normal range. On the 10th day after admission, the patient no longer had fever and was in a clear state of consciousness. The results of the rechecked blood and CSF cultures were negative. Therefore, meropenem was discontinued, and the patient continued to receive a full course of vancomycin as consolidated antibiotic treatment. Vancomycin was discontinued later, after two consecutive negative results of blood culture and CSF culture were obtained, with a total course of vancomycin treatment of 12 days. On the 15th day after admission, the patient’s clinical symptoms had completely resolved. Physical examination: the neck was supple without resistance, muscle strength and tone of all four limbs were normal, meningeal irritation signs were negative, and bilateral pathological reflexes were not elicited. The patient was discharged from the hospital and advised to attend regular follow-up appointments. Telephone follow-ups were conducted 1, 6, 12, and 24 months after discharge, and the patient reported no significant discomfort or adverse symptoms.

## 3 Discussion

*Streptococcus suis* is an important zoonotic pathogen that causes serious economic losses in the pig industry worldwide and poses a potential threat to human health (11). *Streptococcus suis* is a facultatively anaerobic, Gram-positive bacterium. It typically occurs in chains and is non-spore forming, and some strains possess a capsule. It has a large number of serotypes. Currently, 29 serotypes based on capsular polysaccharide antigens have been identified, including SS1–19, 21, 23–25, 27–31, and 1/2, as well as serotype Chz and several novel cps loci (12, 13). Among these, *Streptococcus suis* serotype 2 (SS2) strain is the main cause of infection in pigs and humans worldwide and poses a significant public health challenge (14). The virulence factors of SS2 in serum mainly rely on muramidase-released proteins and extracellular factors, which are two important virulence factors of *Streptococcus suis* type 2 (15). *Streptococcus suis* strains can be genetically classified into sequence types (STs) that appear to exhibit a geographical distribution pattern (16). Globally, ST1 strains are the most frequently isolated from human cases of *Streptococcus suis* infection. In China, two human infection outbreaks caused by the ST7 epidemic strain occurred in Jiangsu Province in 1998 and Sichuan Province in 2005 respectively (17). Its pathogenicity was higher than that of ST1, both leading to high mortality rates. Since 2005, ST1 and ST7 strains have been the primary pathogens responsible for human *Streptococcus suis* infections in China (18).

*Streptococcus suis* often causes severe clinical symptoms such as meningitis, sepsis, and shock, with a mortality rate as high as 10%–30% (19). *Streptococcus suis* meningitis is a type of bacterial meningitis, characterized by acute onset, rapid progression, and severe sequelae, which poses a huge challenge to early clinical diagnosis and treatment. In recent years, with the rapid development of large-scale swine farming, the incidence of *Streptococcus suis* infection has increased. *Streptococcus suis* primarily affects livestock, particularly pigs. It can also be transmitted to humans through contact with infected or carrier pigs via broken skin or mucous membranes, as well as by consuming undercooked pork products (20). In 1968, Perch et al. (21) first reported three human cases of *Streptococcus suis* infection complicated with meningitis and sepsis. Subsequently, epidemic and sporadic cases of human meningitis caused by *Streptococcus suis* have been reported worldwide (22–24). Notably, some patients have no clear history of animal exposure, which complicates efforts to trace the origin of the disease. Furthermore, the clinical manifestations of *Streptococcus suis* meningitis are similar to those of other suppurative meningitides. Routine CSF and biochemical analyses play a key role in differentiating viral, bacterial, tuberculous, and other major categories of neuroinfection but cannot identify a specific causative pathogen. Although blood and CSF cultures are the gold standards for pathogen diagnosis, they are susceptible to the influence of antibiotic treatment and have a long detection cycle, which may lead to diagnostic delay (25). The incubation period for human infection with *Streptococcus suis* varies from several hours to 7 days, with an average of 2–3 days (26). The length of the incubation period is related to factors such as the virulence and quantity of the pathogen, as well as the body’s immunity. Previous studies have confirmed that underlying diseases that can lead to immune dysfunction are important risk factors for *Streptococcus suis* infection (27). This patient had no history of diabetes, chronic liver disease, chronic kidney disease, long-term use of glucocorticoids or immunosuppressants, or any other underlying diseases that could cause immunosuppression. The patient was an individual with normal immune function. The occurrence of *Streptococcus suis* meningitis in this patient indicates that this pathogen has strong virulence. Even in individuals with intact immunity, it can cause severe invasive infections.

In this case, the patient presented with sudden high fever and altered consciousness. Based on the onset time, the incubation period is estimated to be approximately 3 days, which falls within the recognized incubation period range for swine streptococcal infections. The clinical manifestations in this case were consistent with those reported in previous studies. Thanh et al. (5) pointed out in a retrospective analysis of 153 patients with *Streptococcus suis* associated meningitis at the Hospital for Tropical Diseases in Ho Chi Minh City, Vietnam, that the most common manifestation of infection was fever, followed by hearing loss. Deng et al. (6) conducted a clinical manifestation statistics for 17 patients with *Streptococcus suis* meningitis in Liuzhou, China. The most common clinical manifestations were fever, sensorineural hearing loss, headache, and changes in the mental state, which were highly consistent with the symptom characteristics of this case. It is worth noting that in the early stages of the disease, the patient in this case experienced consciousness disorders and restlessness. It is thought that a large number of bacteria multiply in the meninges, triggering a severe inflammatory response. The inflammatory exudate blocks the CSF circulation pathway, causing an imbalance in the production and absorption of CSF, which in turn leads to an increase in intracranial pressure. This finding is consistent with the subsequent examination results. Elevated intracranial pressure further compresses the brain tissue, resulting in ischemia, hypoxia, and impaired nerve cell functions, which in turn causes changes in the patient’s consciousness status.

In terms of diagnosis, neuroimaging findings of *Streptococcus suis* meningitis are mostly normal. Therefore, definitive diagnosis relies on laboratory tests; nevertheless, existing detection methods have certain limitations. Blood and CSF cultures are traditional gold standards, but they are slow and have a relatively low positive rate. The patient was admitted at 23:35. A lumbar puncture was performed the following day at 00:40, during which CSF was collected for bacterial culture, and blood culture tests. *Streptococcus suis* was not detected in the blood or CSF culture until 3 days later (the 5th day after admission). In recent years, next-generation sequencing (NGS) technology has been widely applied in multiple fields, including the genetic identification and traceability of clinical pathogens, surveillance and prevention of emerging infectious diseases and nosocomial infection outbreaks, detection of pathogen drug-resistant gene mutations, and vaccine development (28–30). In this case, to quickly identify the type of pathogen causing intracranial infection, CSF and blood samples were sent to an external laboratory for MetaCAP testing after communicating with the patient’s family. One day later (on the 3rd day after admission), the results indicated a *Streptococcus suis* infection, and this time frame included the sample transportation time. In this case, the adopted MetaCAP technology, a next-generation pathogenic nucleic acid sequencing product, represents a comprehensive upgrade over conventional mNGS, as it incorporates probe capture technolog. By means of “host depletion + million-probe” for pathogen capture, it significantly reduces the interference of host genes in detection, effectively compensates for the deficiencies of traditional mNGS technology in the sensitivity and specificity of pathogen, drug-resistant gene, and virulence gene detection, and increases the pathogen detection rate by at least 10%–15% compared with mNGS. For pathogens causing central nervous system infections, such as *Streptococcus suis*, MetaCAP shortens the detection time from the 3–5 days needed for traditional culture to just a few hours, significantly improving the efficiency of early diagnosis.

The core of treatment for *Streptococcus suis* meningitis is to rapidly control the infection and reduce the damage to the central nervous system. However, the key challenge in treatment is the blood-brain barrier (BBB). Most antibiotics struggle to cross the BBB and enter the CSF, resulting in insufficient drug concentrations in the CSF. Therefore, for treatment before the return of drug susceptibility test results, third-generation cephalosporins can be used as an empirical treatment plan (31). If the patient has one or more risk factors for Listeria monocytogenes infection, or if third-generation cephalosporins are not immediately available, ampicillin, amoxicillin, or penicillin G can be added to the initial bacterial treatment plan (31). With the widespread use of antibacterial drugs, drug resistance is becoming increasingly prominent. In some regions, the sensitivity of bacterial strains to penicillins and cephalosporins has decreased, and even multi-drug-resistant strains have emerged. In such cases, vancomycin can be administered intravenously (32–34). Vancomycin is a potent glycopeptide that acts as an antibacterial agent. Its main mechanism of action is to bind to the D-alanyl-D-alanine (D-Ala-D-Ala) terminus of bacterial cell wall precursor molecules, block cross-linking of the peptidoglycan layer, and inhibit the synthesis of bacterial cell walls to kill bacteria. It is particularly effective against Gram-positive bacteria (10, 35). Previous studies have confirmed that most strains of *Streptococcus suis* are sensitive to vancomycin (5, 8, 36–40). Furthermore, there have been previous studies used vancomycin for treatment (41–43). However, as a macromolecular glycopeptide antibiotic, it is extremely difficult to cross the intact BBB under normal circumstances. When central nervous system infections, such as meningitis, increase the permeability of the BBB, it can reach a certain concentration in the CSF and exert its antibacterial effect (44, 45). The patient in this case had received cephalosporin treatment at a previous hospital, but the therapeutic effect was poor, and drug resistance could not be ruled out. Given the patient’s severe condition, meropenem was initially administered. After the diagnosis was confirmed, vancomycin was added for treatment. The results of the drug sensitivity test indicated that vancomycin was sensitive to infection. This result further verified the rationality of our subsequent use of vancomycin and the adjustment of the dosing regimen. Notably, in this case, intrathecal injection of vancomycin was administered as part of the treatment. For the clinical treatment of *Streptococcus suis* meningitis, intrathecal injection of antibiotics is not the first-line routine treatment. However, in critically ill patients, this therapeutic approach addresses the difficulty in crossing the BBB and achieving insufficient intracranial drug concentrations after intravenous administration, thereby enhancing the local antibiotic treatment effect. Therefore, after a joint assessment by a multidisciplinary team, we added intrathecal injection of vancomycin to the treatment regimen. The patient in this case had a good prognosis and did not develop sequelae such as cognitive impairment or hearing loss. Considering that the patient received antibiotic treatment within 24 h of symptom onset, and the treatment regimen was adjusted promptly based on drug susceptibility results with the additional use of dexamethasone, the risk of disability was reduced to a certain extent. However, long-term follow-up is necessary to assess neurological function recovery.

## 4 Strengths and limitations

This case report provides a complete record of the entire treatment process for a critically ill patient with *Streptococcus suis* meningitis. The clinical data is comprehensive and detailed. We confirmed the pathogen through both CSF culture and molecular testing. At the same time, we proposed an innovative treatment plan for intrathecal administration of vancomycin, which provided valuable practical references for the clinical management of similar severe cases. However, the results of this study were based on a single patient and have limited extrapolation potential. We need larger sample size observational studies or clinical trials to further verify the universality of the aforementioned treatment strategy. The drug sensitivity test did not include meropenem, which limited the comprehensive assessment of potential antibiotic options for this strain. Moreover, the lack of monitoring of serum and CSF concentrations of vancomycin is also a limitation.

## 5 Conclusion

Currently, research on *Streptococcus suis* meningitis mainly focuses on epidemiological investigations and pathogenesis exploration. In-depth analyses of specific clinical cases remain relatively rare. This article reports a typical case of *Streptococcus suis* meningitis. It provides a detailed description of clinical features, diagnostic process, treatment plan, and prognosis. This study aims to improve clinicians’ understanding of this disease. It also provides a reference basis for early identification and standardized treatment.

## 6 Patient perspective

On the 7th day of anti-infection treatment with vancomycin, the patient’s consciousness gradually cleared. When providing feedback on the treatment, the patient said, “In the early stage of the illness, my thoughts were very confused, I couldn’t control my emotions, and I had severe dizziness. After this period of treatment, my dizziness gradually relieved, and my mood improved significantly.” On the day of discharge, the patient said, “All my previous discomforts have completely gone, and I’m doing great now.” During the telephone follow-up at 3 months after discharge, the patient said, “I have no discomforts at all, and my quality of life hasn’t been affected.”

## Data Availability

The raw data supporting the conclusions of this article will be made available by the authors, without undue reservation.
